# Dual and pan-peroxisome proliferator-activated receptors (PPAR) co-agonism: the bezafibrate lessons

**DOI:** 10.1186/1475-2840-4-14

**Published:** 2005-09-16

**Authors:** Alexander Tenenbaum, Michael Motro, Enrique Z Fisman

**Affiliations:** 1 Cardiac Rehabilitation Institute, Sheba Medical Center, 52621 Tel-Hashomer, Israel; 2 Sackler Faculty of Medicine, Tel-Aviv University, 69978 Tel-Aviv, Israel; 3 Cardiovascular Diabetology Research Foundation, 58484 Holon, Israel

## Abstract

There are three peroxisome proliferator-activated receptors (PPARs) subtypes which are commonly designated PPAR alpha, PPAR gamma and PPAR beta/delta. PPAR alpha activation increases high density lipoprotein (HDL) cholesterol synthesis, stimulates "reverse" cholesterol transport and reduces triglycerides. PPAR gamma activation results in insulin sensitization and antidiabetic action. Until recently, the biological role of PPAR beta/delta remained unclear. However, treatment of obese animals by specific PPAR delta agonists results in normalization of metabolic parameters and reduction of adiposity. Combined treatments with PPAR gamma and alpha agonists may potentially improve insulin resistance and alleviate atherogenic dyslipidemia, whereas PPAR delta properties may prevent the development of overweight which typically accompanies "pure" PPAR gamma ligands. The new generation of dual-action PPARs – the glitazars, which target PPAR-gamma and PPAR-alpha (like muraglitazar and tesaglitazar) are on deck in late-stage clinical trials and may be effective in reducing cardiovascular risk, but their long-term clinical effects are still unknown. A number of glitazars have presented problems at a late stage of clinical trials because of serious side-effects (including ragaglitazar and farglitazar). The old and well known lipid-lowering fibric acid derivative bezafibrate is the first clinically tested pan – (alpha, beta/delta, gamma) PPAR activator. It is the only pan-PPAR activator with more than a quarter of a century of therapeutic experience with a good safety profile. Therefore, bezafibrate could be considered (indeed, as a "post hoc" understanding) as an "archetype" of a clinically tested pan-PPAR ligand. Bezafibrate leads to considerable raising of HDL cholesterol and reduces triglycerides, improves insulin sensitivity and reduces blood glucose level, significantly lowering the incidence of cardiovascular events and new diabetes in patients with features of metabolic syndrome. Clinical evidences obtained from bezafibrate-based studies strongly support the concept of pan-PPAR therapeutic approach to conditions which comprise the metabolic syndrome. However, from a biochemical point of view, bezafibrate is a PPAR ligand with a relatively low potency. More powerful new compounds with pan-PPAR activity and proven long-term safety should be highly effective in a clinical setting of patients with coexisting relevant lipid and glucose metabolism disorders.

## Peroxisome proliferator-activated receptors

Peroxisome proliferator-activated receptors (PPARs) are nuclear hormone receptors, i.e. ligand-dependent intracellular proteins that stimulate transcription of specific genes by binding to specific DNA sequences following activation by the appropriate ligand. When activated, the transcription factors exert several functions in development and metabolism. There are three PPAR subtypes which are the products of distinct genes and are commonly designated PPAR alpha, PPAR gamma and PPAR beta/delta, or merely delta [1-4]. The PPARs usually heterodimerize with another nuclear receptor, the 9-*cis*-retinoic acid receptor (RXR), forming a complex that interacts with specific DNA response elements within promoter regions of target genes. When activated by agonist ligand binding, this heterodimer complex recruits transcription coactivators and regulates the transcription of genes involved in the control of lipid and carbohydrate metabolism [1-4].

*PPAR alpha*, activated by polyunsaturated fatty acids and fibrates, is implicated in regulation of lipid metabolism, lipoprotein synthesis and metabolism and inflammatory response in liver and other tissues. PPAR alpha is highly expressed in tissues with high fatty acid oxidation (like liver, kidney and heart muscle), in which it controls a comprehensive set of genes that regulate most aspects of lipid catabolism. Like several other nuclear hormone receptors, it heterodimerizes with RXR alpha to form a transcriptionally competent complex [1-3,5]. In addition, PPAR-alpha is expressed in vascular endothelial cells, smooth muscle cells, monocyte/macrophages and T lymphocytes. PPAR alpha activation increase HDL cholesterol synthesis, stimulate "reverse" cholesterol transport and reduce triglycerides [1-3,6].

*PPAR gamma *plays important roles in the regulation of proliferation and differentiation of several cell types, including adipose cells. It has the ability to bind a variety of small lipophilic compounds derived from both metabolism and nutrition. These ligands, in turn, determine cofactor recruitment to PPAR gamma, regulating the transcription of genes in a variety of complex metabolic pathways. PPAR gamma is highly expressed in adipocytes, where it mediates differentiation, promotes lipid storage, and, as a consequence, is thought to indirectly improve insulin sensitivity and enhance glucose disposal in adipose tissue and skeletal muscle [7-9]. Its activation by drugs of the glitazones (thiazolidinediones) group results in insulin sensitization and antidiabetic action.

Until recently, the biological role of *PPAR delta *remained unclear. Animal studies revealed that PPAR delta play an important role in the metabolic adaptation of several tissues to environmental changes. Treatment of obese animals by specific PPAR delta agonists results in normalization of metabolic parameters and reduction of adiposity. PPAR delta appeared to be implicated in the regulation of fatty acid burning capacities of skeletal muscle and adipose tissue by controlling the expression of genes involved in fatty acid uptake, beta-oxidation and energy uncoupling. PPAR delta is also implicated in the adaptive metabolic response of skeletal muscle to endurance exercise by controlling the number of oxidative myofibers, inducing so and enhancing fatty acid catabolism in muscular tissue [3,6,10]. Moreover, recent studies revealed that ligand activation of these receptors is associated with improved insulin sensitivity and elevated HDL levels thus demonstrating promising potential for targeting PPAR delta in the treatment of obesity, dyslipidemias and type 2 diabetes [11].

## Clinical studies of PPAR ligands

Fibric acid derivatives (fibrates) are PPAR alpha ligands. Fibrates have been used in clinical practice for more than four decades as a class of agents known to decrease triglyceride levels while substantially increasing HDL-cholesterol levels, with a limited but significant additional lowering effect on low density lipoprotein (LDL)-cholesterol levels [5]. In addition to their favorable effects on lipid profiles, evidence is mounting that benefits may also stem from the anti-inflammatory and antiatherosclerotic properties of these drugs [12,13]. Although fibrate trials have reported cardiovascular risk reduction in patients with dyslipidemia, it is evident that the favorable alterations in plasma lipids can only partially explain the reduction in cardiovascular events in these studies. This is particularly evident for high-risk individuals, such as diabetics or patients with insulin resistance who may have more pronounced cardiovascular benefits [5,12-15].

Glitazones are synthetic PPAR gamma ligands with well recognized effects on glucose and lipid metabolism. The clinical use of these PPARgamma agonists in type 2 diabetic patients leads to an improved glycemic control and an inhanced insulin sensitivity, and – at least in animal models – to a protective effect on pancreatic beta-cell function. Glitazones may also have cardiovascular benefits. Animal models of atherosclerosis have shown that these drugs reduce the extent of atherosclerotic lesions and inhibit macrophage accumulation. Clinical studies have also shown that these drugs improve the lipid profile of patients at risk of developing atherosclerosis and reduce circulating levels of inflammatory markers [16-18]. However, they can produce adverse effects, generally mild or moderate, but some of them (mainly peripheral edema and weight gain) may lead to treatment cessation.

Currently, clinical studies regarding PPAR delta ligands are lacking. Given the results obtained with animal models, PPAR delta agonists may have therapeutic usefulness in metabolic syndrome by increasing fatty acid consumption in skeletal muscle and adipose tissue [19]. Probably, weight reduction could be expected as well.

## Dual and pan-PPAR co-agonism

Combined treatments with PPAR gamma and alpha agonists may potentially improve insulin resistance and alleviate atherogenic dyslipidemia, whereas PPAR delta properties may prevent the development of overweight which typically accompanies "pure" PPAR gamma ligands like glitazones. With extended use, it is hoped that these effects will reduce the risk of long-term cardiovascular complications. PPAR alpha and gamma stimulation play complementary roles in the prevention of atherosclerosis. Cholesterol accumulation in macrophages located in the endothelium is a crucial step in the formation of atherosclerosis. PPAR gamma activation is necessary for the efflux of cholesterol from macrophage foam cells. Cholesterol taken up by HDL particles containing apolipoportein A-1 is transported to the liver to be disposed of as bile acids [3,15,17]. PPAR alpha agonists, on the other hand, speed up the transfer of cholesterol from macrophages to particles containing apolipoportein A-1 [3,16,20].

Thus, compounds with dual PPAR alpha/PPAR gamma activity appear well-suited for the treatment of diabetic patients with the additional risk factor of dyslipidemia. The finding that PPAR agonists play a role in regulating other processes, such as inflammation, vascular function, and vascular remodeling, has highlighted further potential indications for these agents [16,17]. So far, therefore, a relatively high number of dual PPAR alpha and PPAR gamma agonists have been described [3,21-25]. The new generation of dual-action PPARs – the glitazars which target PPAR-gamma and PPAR – alpha (muraglitazar and tesaglitazar) are on deck in late-stage clinical trials and may be effective in reducing cardiovascular risk, but their long-term clinical effects are still unknown. A number of glitazars have problems in late stage clinical trials because of serious side-effects (including ragaglitazar and farglitazar).

## The bezafibrate lessons: feasibility of dual and pan-PPAR co-agonism in a clinical setting

The old and well known lipid-lowering fibric acid derivative bezafibrate is the first clinically tested pan – (alpha, beta/delta, gamma) PPAR activator [26-33]. It is a sole pan PPAR activator with more than a quarter of a century of a therapeutic experience with a good safety profile. Therefore, bezafibrate could be considered (indeed, as a "post hoc" understanding) as an "archetype" of a clinically tested pan-PPAR ligand. In patients with relevant metabolic abnormalities it is expected to improve both insulin sensitivity and the blood lipid profile and probably reduce the risk of long-term cardiovascular complications. In addition, we can expect prevention of overweight development due to its PPAR-beta/delta properties.

So, which are the data regarding bezafibrate administration? In a large trial in 1568 men with lower extremity arterial disease, bezafibrate reduced the severity of intermittent claudication for up to three years [34]. In general, the incidence of coronary heart disease in patients on bezafibrate has tended to be lower, but this tendency did not reach statistical significance. However, bezafibrate had significantly reduced the incidence of non-fatal coronary events, particularly in those aged <65 years at entry, in whom all coronary events may also be reduced [34]. In two other independent studies bezafibrate decreased the rate of progression of coronary atherosclerosis and decreased coronary events rate [35,36]. In the the Bezafibrate Infarction Prevention (BIP) study an overall trend of a 9.4% reduction of the incidence of primary end point (fatal or non-fatal myocardial infarction or sudden death) was observed. The reduction in the primary end point in 459 patients with high baseline triglycerides (200 mg/dL or more) was significant [37].

Our new data demonstrate that bezafibrate can significantly reduce the incidence of myocardial infarction (MI) in patients with metabolic syndrome [38]. The decrease in MI incidence among patients on bezafibrate was reflected in a trend to a late risk reduction of cardiac mortality during a long-term follow-up period. This tendency was strengthened in patients with augmented features (at least 4 risk factors for metabolic syndrome) of metabolic syndrome (56% reduction of cardiac mortality during 8-year follow-up). It is interesting that in patients without metabolic syndrome this favorable effect was not presented: There was no significant difference in the cardiovascular end-points between bezafibrate and placebo groups.

Previous observations have shown beneficial effects of bezafibrate on glucose and insulin metabolism [39-41]. Recently, we have shown thata pharmacological intervention with bezafibrate decreased the incidence and delayed the onset of type 2 diabetes in patients with impaired fasting glucose levels, and in obese patients over a long-term follow-up period [42,43]. In the BIP study the rates of adverse events were similar in both study groups [37]. Thus, bezafibrate treatment was safe in addition to being effective in diabetes prevention. Moreover, there was no significant change in mean body mass index values in either the bezafibrate or the placebo group during the follow-up [38,42,43].

Therefore, the pan – (alpha, beta, gamma) PPAR activator bezafibrate leads to a considerable raising of HDL cholesterol and a reduction of triglycerides, improves insulin sensitivity and reduces blood glucose level, significantly lowering the incidence of cardiovascular events and new diabetes in patients with features of metabolic syndrome over a long-term follow-up period. We conclude that clinical evidences obtained from bezafibrate-based studies strongly support the concept of a pan-PPAR therapeutic approach to conditions which comprise the metabolic syndrome. However, from a biochemical point of view, bezafibrate is PPAR ligand with a relatively low potency. We believe that more powerful compounds with pan-PPAR activity and proven long-term safety should be highly effective in a clinical setting of patients with coexisting relevant lipid and glucose metabolism disorders.

## List of abbreviations used

BIP – Bezafibrate Infarction Prevention

HDL – high density lipoprotein

LDL – low density lipoprotein

MI – myocardial infarction

PPAR – peroxisome proliferator-activated receptor

RXR – retinoic acid receptor

## Competing interests

The author(s) declare that they have no competing interests.

## Authors' contributions

All authors have equally contributed in the conception and drafting of the manuscript.
